# Event- and biostratigraphic evidence for two independent Ries and Steinheim asteroid impacts in the Middle Miocene

**DOI:** 10.1038/s41598-022-21409-8

**Published:** 2022-11-03

**Authors:** Elmar Buchner, Volker J. Sach, Martin Schmieder

**Affiliations:** 1 HNU - Neu-Ulm University of Applied Sciences, Wileystraße 1, 89231 Neu-Ulm, Germany; 2 Meteorkrater-Museum Steinheim, 89555 Steinheim am Albuch, Germany; 3 Fokus Natur, Am Heselsberg 29, 88416 Ochsenhausen, Germany

**Keywords:** Palaeontology, Sedimentology, Seismology, Natural hazards, Planetary science

## Abstract

For decades, the Nördlinger Ries and Steinheim Basin in southern Germany have been regarded as a textbook example of a terrestrial impact crater doublet, although the oldest crater lake deposits in both craters suggest a biostratigraphic age difference of ~ 0.5 to 1 Myr. We previously presented stratigraphic arguments that challenged the double impact scenario and favoured a model of two temporally independent impact events in the Mid-Miocene. We here present, for the first time, four localities within a distance of ~ 50–100 km from the Ries and ~ 50–70 km from the Steinheim crater that expose two independent seismite horizons, together unique within the Upper Freshwater Molasse of the North Alpine Foreland Basin, each one featuring impressive water escape structures. The seismite horizons are separated by ~ 10 to 15 m of undisturbed Molasse deposits and, biostratigraphically, by an entire European Land Mammal Zone, thus providing evidence for two independent major seismic events within a time span of ~ 0.5–1 Myr. Both the lower and the upper seismite horizons can be correlated litho- and biostratigraphically with the basal crater lake sediments at the Ries and Steinheim craters, respectively, deposited immediately after the impacts. From a biostratigraphic point of view, the impact event that formed the Steinheim Basin probably occured around 14 Ma, some 0.8 Myr after the ~ 14.81 Ma Ries impact event.

## Introduction

**Figure 1:**
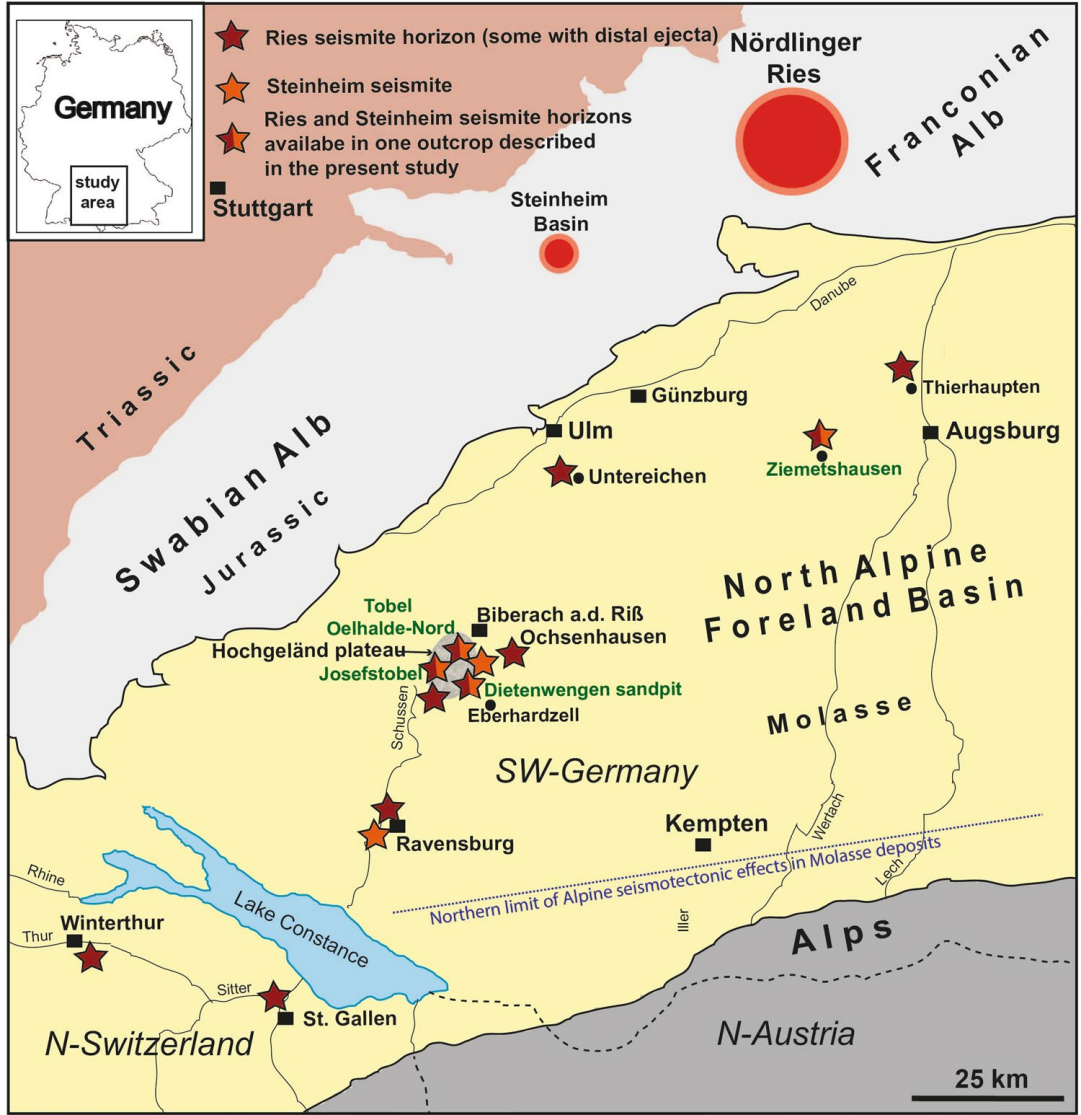
Geographic and geologic situation in the study area in southern Germany, northern Switzerland and Austria. Marked by red stars are outcrops of the Ries seismite within a distance of up to 205 km from the Ries crater, and the proposed Steinheim seismite (orange stars) within a distance of up to 100 km from the Steinheim crater. Four localties in green are presented and discussed in this study and are marked with stars half in red and orange. Note that no seismite occurences caused by Alpine seismotectonic events are known North of the dotted line.

The ~ 24 km-diameter Nördlinger Ries and the ~ 4 km-diameter Steinheim Basin are two well-preserved Miocene impact craters in the karstified Swabian-Franconian Alb limestone plateau of southern Germany^1–3^ (Fig. 1). The complex Ries crater is characterized by a double-layered ejecta blanket^4^ composed of lithic impact breccia derived mainly from weakly shocked Jurassic to Triassic sedimentary target rocks, as well as by the overlying suevite that contains variably shocked and partly impact-melted material derived from the crystalline crater basement. High-precision Ar–Ar results suggest the Nördlinger Ries impact occurred at ~ 14.81 Ma^5–7^.

The ~ 4 km-diameter Steinheim Basin, located about 40 km SW of the centre of the Ries crater, is a complex impact crater with a prominent central uplift within a sequence of Triassic and Jurassic sedimentary rocks^1,2^. The Steinheim Basin is well known for its shatter cones of outstanding shape and quality^1,8^. Numerous drillings into the Steinheim Basin yielded cores with the basin-filling impact breccia overlain by crater lake sediments^1^. The impact breccia contains variable amounts of clasts of Jurassic limestones, marls, mudstones, and sandstones^9^, while the post-impact sediments are particularly rich in fossils, most notably gastropods, ostracods and vertebates such as fishes, birds, turtles, reptiles, land mammals, and plants^1,10–12^. Although isotopic dating has, thus far, failed to yield a geologically meaningful age for the Steinheim Basin, the impact event is widely thought to have formed simultaneously with the Nördlinger Ries crater^13^.

Beside the impact ejecta inside and in the surroundings of the two craters, a conspicuous bed of coarse-grained distal ejecta in the North Alpine Foreland Basin (henceforth NAFB) represents an important impact-marker horizon^3^. This distal Ries ejecta layer (locally known as the ‘Brockhorizont’) occurs as a locally reworked horizon of sand including shocked quartz grains, pebbles, cobbles, and boulders of predominantly Upper Jurassic limestone with rare shatter cones^14–18^. The lithic components in this horizon were ejected during the Ries impact event and ballistically transported over distances as long as 180 km (~ 7 to 8 crater diameters^19^) and preserved in the siliciclastic sediments of the NAFB^15,17,18^. No such coarse-grained ejecta have thus far been reported from the much smaller Steinheim impact. In theory, the most distal ejecta components of recognisable size (i.e., measuring at least several millimeters across) would be expected within a distance of less than 30 km from the impact site^20^, i.e., they would probably not have reached the NAFB. The average thickness of a Steinheim ejecta layer in the Hochgeländ area, about 100 km south of the Steinheim crater, would be expected to be approximately one millimeter, or less^21^, and would mainly contain grain sizes of sand and smaller, perhaps with occasional larger ejecta particles. Another factor hampering the recognition and stratigraphic correlation of Steinheim ejecta may be the internal structure of the karstified, highly porous Upper Jurassic target rock, probably in combination with some post-impact erosion. The high target rock porosity may have suppressed the primary production of proximal (and medial) impact ejecta in the closer surroundings of the Steinheim Basin in the first place^22^.

One effect of large asteroid impacts is, depending on the kinetic energy imparted into the target rock, the triggering of intense earthquakes^20,21,23^. The extremely powerful Chicxulub impact, known to have terminated almost all Mesozoic life, is thought to have generated a seismic pulse roughly equivalent to a magnitude M_W_ (moment magnitude scale) ~ 10 to 11.5 earthquake^24,25^. The impact that formed the 24 km-diameter Ries crater likely caused an earthquake around magnitude M_W_ ~ 8.5^3,26,27^. The earthquake triggered by the much smaller Steinheim impact event was presumably stronger than M_W_ ~ 6.4 and may have been as strong as magnitude M_W_ ~ 7 or even somewhat higher^3^.

Impact-triggered earthquakes can produce seismites in extensive volumes of sediment^3,27,28^. One form of impact-related seismites are clastic dikes that commonly occur more or less proximal to the corresponding impact sites^29^. However, impact-earthquakes may also cause soft-sediment deformation through liquefaction at greater distances (hundreds of kilometres) from the point of impact^3,27,30^. The exact style of seismically induced deformation is mostly governed by the composition and lithification of the surface-near sediments and their petrophysical properties (i.e., cementation/diagenesis, grain size, open pore space, water saturation^3,27^).

Both the Nördlinger Ries and the Steinheim asteroid impact events imparted a significant amount of the impact energy into the sedimentary target of the Swabian-Franconian Alb, causing regional-scale disturbances^3,16,21,27,31^. An extensive impact-seismite unit marks the Ries impact in the statigraphic record within the sand-dominated Upper Freshwater Molasse (‘Obere Süßwassermolasse’, OSM), with a number of representative outcrops in the vicinity of Biberach an der Riß, Ochsenhausen, and Ravensburg^3,27^. The Ries seismite is capped by a primary (in situ-preserved) horizon of distal impact ejecta and is, in turn, overlain by undisturbed OSM deposits^15,16^, a telltale sign that the seismite is the product of a major earthquake triggered by the Ries event. At Biberach and Ravensburg, swarms of clastic dikes cut through the Ries-related seismite-ejecta couplet and portions of the overlying OSM^16^, which we interpret as evidence for a second, high-magnitude earthquake in the region that occurred subsequent to the Ries earthquake.

For decades, the Nördlinger Ries and the Steinheim Basin had been widely considered as a terrestrial impact crater doublet^13,32^, formed by the impact of a binary asteroid of ~ 1 km (Ries impactor) and ~ 100–150 m (Steinheim impactor) in diameter, respectively^13^. However, the long-held double-impact theory is, based on palaeontologic and structural geologic constraints, not fully supported^3,12,33^ (and discussions and references therein). From a biostratigraphic point of view, the Steinheim impact may postdate the Ries event by several kyr, and potentially as much as 1 Myr^1^. Accordingly, we recently suggested the two impact structures are likely of different age, supported by biostratigraphic, sedimentologic, and event stratigraphic evidence^3^. While the basal crater lake sediments within the ~ 14.81 Ma Ries crater^5,6^ correspond to the transition from the European land mammal zones (ELMZ) MN 5 to MN 6 (Langhian), the oldest crater lake deposits at Steinheim fall in the transition zone of higher MN 6 to MN 7 (latest Langhian and Serravallian^3,12,34,35^).

Although an at least ~ 17 m-long clastic dike crosscutting the Ries seismite and the overlying distal Ries ejecta horizon in the Tobel Oelhalde-Nord (Hochgeländ plateau near Biberach) was tentatively linked to seismic shaking triggered by the the Steinheim impact^16^, some doubt remained with respect to the impact-earthquake origin of the dike. First, the upper end of the dike is not (unlike the Ries seismite capped by distal Ries ejecta) overlain by a diagnostic Steinheim-impact layer, as is expected at the long distance from the relatively small source crater. Second, the Biberach clastic dike, by its structural nature, cannot be easily correlated laterally with the subhorizontal deposits of the Ries seismite and overlying deposits, and generally, both the dike attributed to the Steinheim-earthquake and the Ries seismite occur as far as ~ 100 km from the two impact structures.

In addition to an earlier detailed description and interpretation of the outcrop Tobel Oelhalde-Nord, we here present three other localities with newly discovered outcrops in the NAFB within a distance of 53 to 100 km from the two impact craters where two separate seismite horizons, both unique to the otherwise more or less undisturbed OSM sediments, are preserved. All seismite outcrops are discussed with respect to their potential relation to the Middle Miocene Ries and Steinheim meteorite impact events in southern Germany, reinforcing the notion that the Steinheim impact may have triggered a significant earthquake several kyr after the Ries-earthquake. In the context of a related earlier publication of our working group^3^, the most important innovation in this follow-up study refers to the biostratigraphic context of the fossil content in deposits below and above the seismite horizons within the newly described outcrops and the biostratigraphically determined relative age of the crater lake deposits in the Ries and the Steinheim impact structures.

## Results

### Outcrops with two seperate seismite horizons

**Figure 2:**
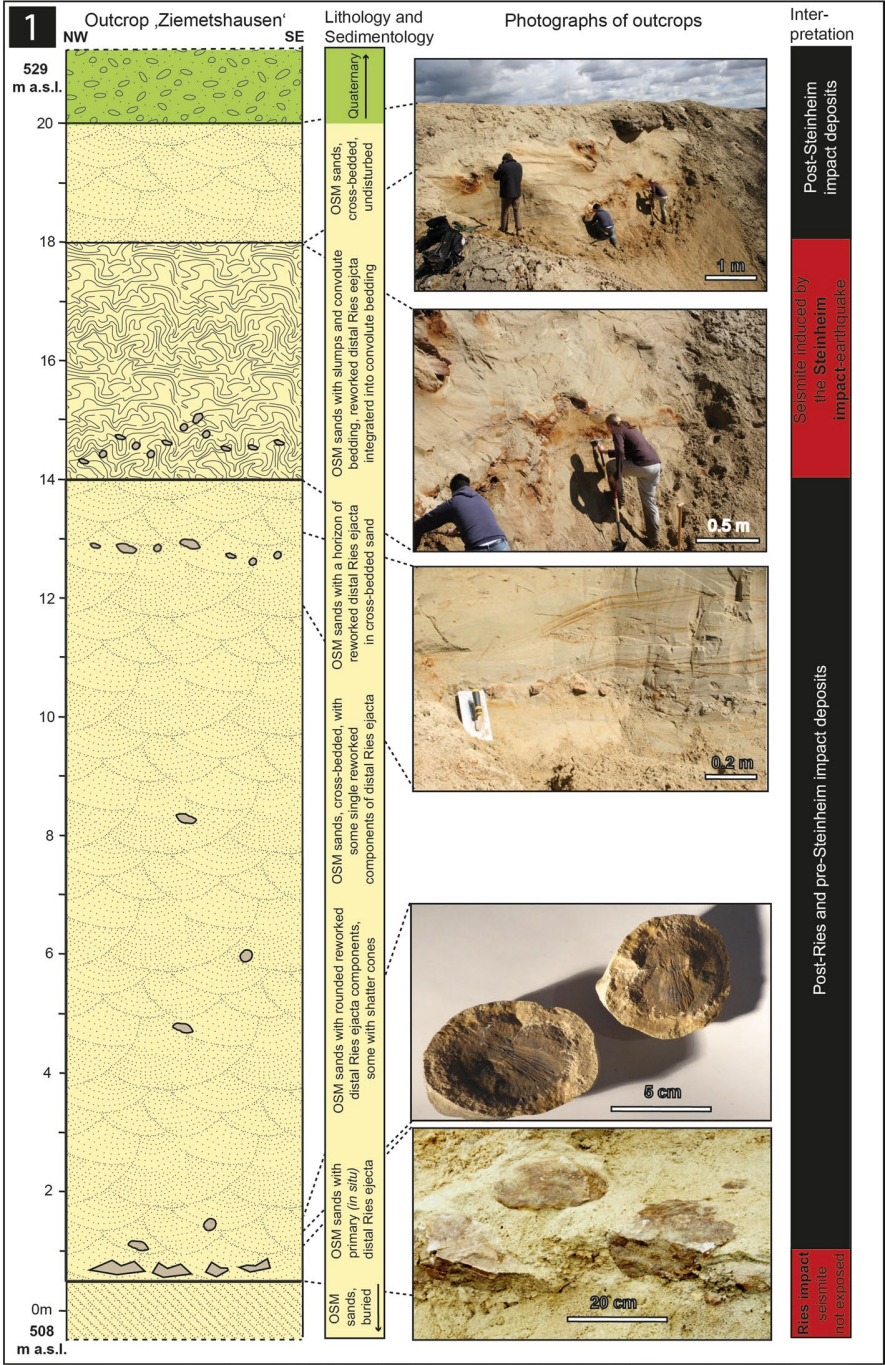
Schematic description of the sedimentological situation (left), outcrop photographs, and event stratigraphic interpretion of the sedimentologic succession (right) in the (former) sand pit Ziemetshausen; outcrop no. 1 in the text. Two impact-seismic events (Ries and Steinheim? impact-earthquakes) are reported by the occurrence of a primary horizon of distal Ries ejecta. Due to the mining level in that outcrop, only the top of sediments of the lower seismite horizon with indistinct convolute bedding were observed. A metre-thick seismite horizon underlying the distal Ries ejecta was not detected unequivocally in this sand pit, but is to be expected. The second impact-earthquake (Steinheim impact) induced a relatively thick seismite horizon with slumps and convolute bedding. A succession of about 12 m of undisturbed OSM sediments were deposited between the two impact events. Photographs of the Steinheim seismite (upper three pictures) were taken by E.B. in 2008 and those of the distal Ries ejecta (lower two pictures) by Peter Bockstaller (Schopfheim) in 2005.

**Figure 3:**
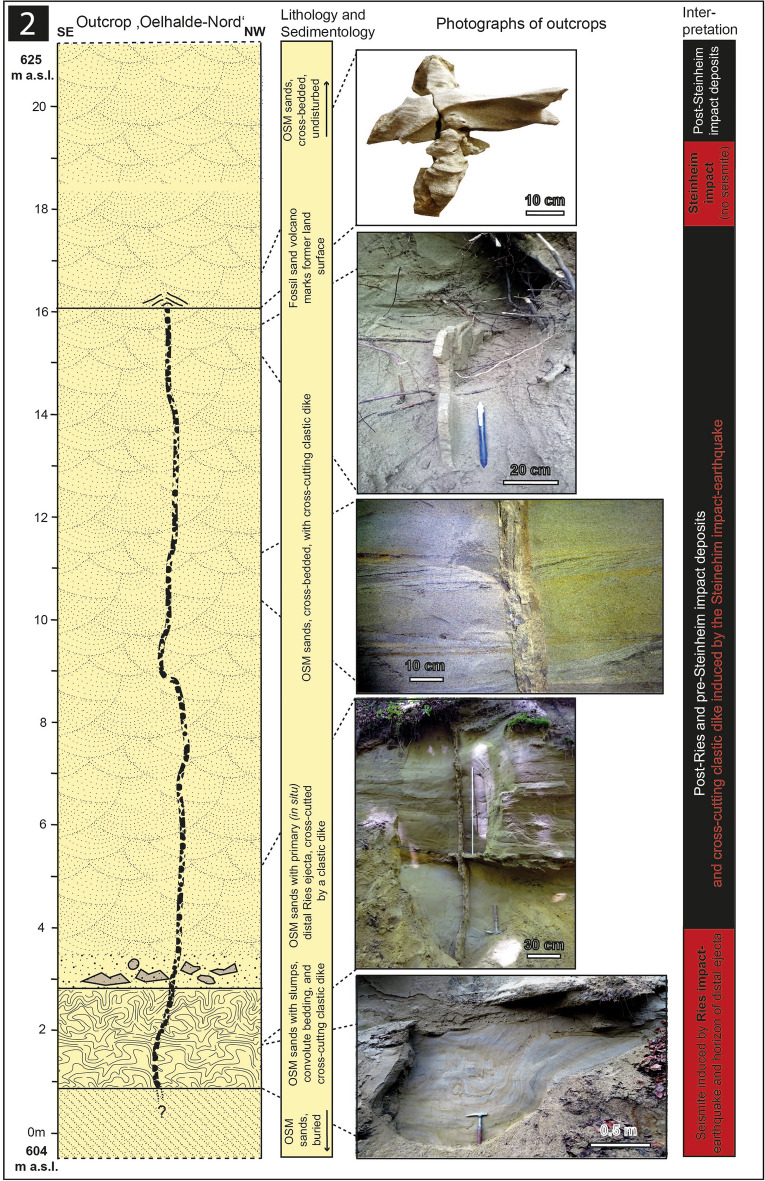
Schematic description of the sedimentological situation (left), outcrop photographs, and event stratigraphic interpretion of the sedimentologic succession (right) in the Tobel Oelhalde-Nord (Hochgeländ plateau south of Biberach an der Riß; outcrop no. 2 in the text). Two seismic events (Ries and Steinheim impact-earthquakes) are reported by the occurrence of the Ries seismite horizons with slumps and convolute bedding within sediments that formed the land surface at the time of the impact event, capped by distal Ries ejecta. The second impact-earthquake (Steinheim impact) caused the formation of a giant clastic dike that reached the palaeo-surface at the time of the second impact event. A succession of about 15 m of OSM sediments were deposited in the timespan between the two impact events; for more details about this outcrop see^16^; all photographs taken by V.J.S. in 2018–2019.

**Figure 4:**
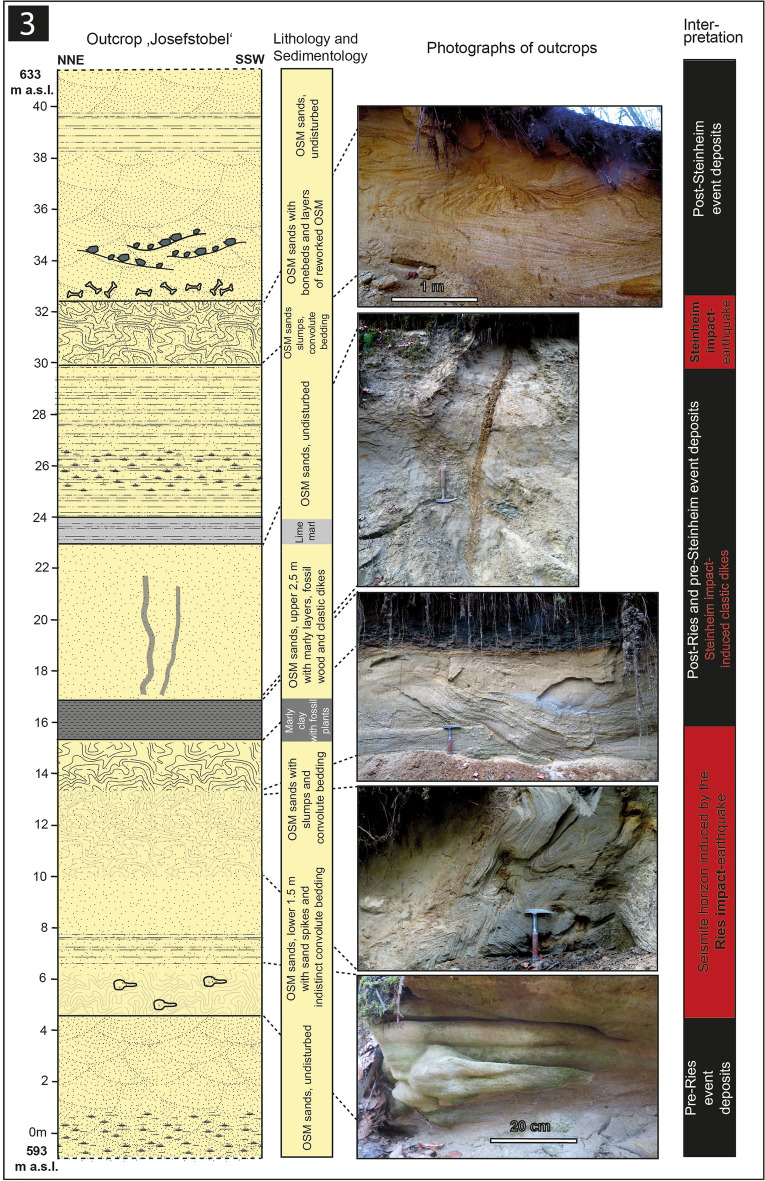
Schematic description of the sedimentological situation (left), outcrop photographs, and event stratigraphic interpretion of the sedimentologic succession (right) in the Josefstobel (Hochgeländ plateau south of Biberach an der Riß; outcrop no. 3 in the text). Two seismic events (Ries and Steinheim impact-earthquakes) are reported by the occurrence of two independent seismite horizons with slumps and convolute bedding within sediments that formed the land surface at the time of the two impact events. Additionally, the Steinheim impact-earthquake caused the formation of clastic dikes in deeper parts of the affected sediments. Usually, the Ries seismite is capped by distal Ries ejecta in several outcrops in the Hochgeländ area and elsewhere. In the Josefstobel, the distal Ries ejecta horizon is substituted by a dark claystone layer that resembles the K-Pg boundary layer to a certain degree (all photographs taken by V.J.S. in 2021).

**Figure 5:**
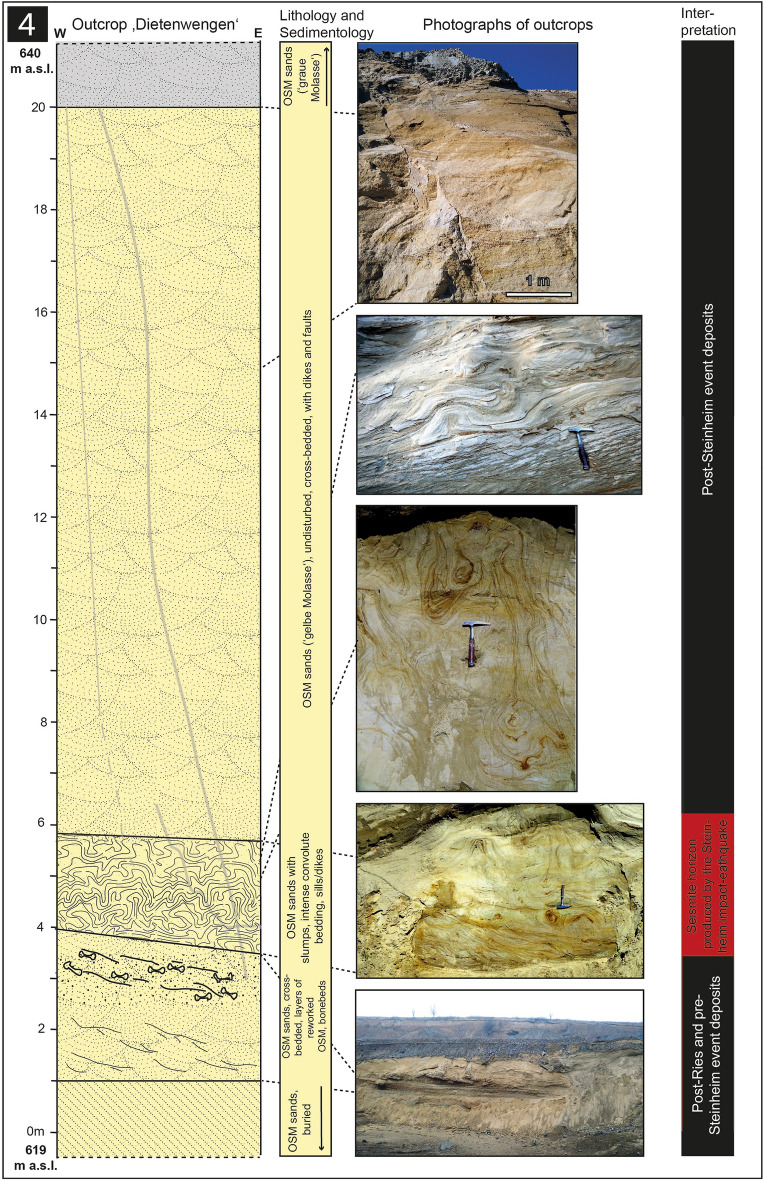
Schematic description of the sedimentological situation (left), outcrop photographs, and event stratigraphic interpretion of the sedimentologic succession (right) in the Dietenwengen sand and gravel pit (near Eberhardzell, ~ 2 km east of the Hochgeländ plateau; outcrop no. 4 in the text). In this outcrop, the effects of the seismic event linked with the Steinheim impact are represented by slumps, convolute bedding, and ball-and-pillow structures within the upper seismite unit (all photographs taken by V.J.S. in 2021).

We here report on four outcrops: 1. The Ziemetshausen sand pit, ~ 53 km S of the rim of the Ries crater and ~ 53 km SE of the Steinheim Basin^36^ (outcrop no. 1; Figs. 1, 2). In this outcrop, the uppermost part of the lower seismite horizon capped by distal Ries ejecta and the upper seismite horizon are excellently exposed and described here for the first time. 2. The Tobel Oelhalde-Nord^3,16,27^ (outcrop no. 2; Figs. 1, 3) located ~ 100 to 105 km South of the Ries crater and ~ 70 km South of the Steinheim Basin. From this outcrop described previously^3,16^, we present updated information that underlines that two independent large seismic events are recorded within NAFB deposits, although no upper seismite horizon occurs in this outcrop. 3. The Josefstobel (outcrop no. 3; Figs. 1, 4), a ravine located in in the Hochgeländ plateau south of Biberach an der Riß, ~ 100 to 105 km South of the Ries crater and ~ 70 km South of the Steinheim Basin, where the lower and the upper seismite horizons (the latter is first reported in the present study) are exposed exceptionally well, and 4. The Dietenwengen sand and gravel pit some kilometers East of the Hochgeländ (outcrop no. 4; Figs. 1, 5), ~ 105 km South of the Ries crater and 70 km South of the Steinheim Basin. This active sand pit offers outstanding insights into sedimentologic features and the biostratigraphy of the upper seismite horizon, also reported for the first time in this study.

### Outcrop no. 1: Ziemetshausen

**Figure 6:**
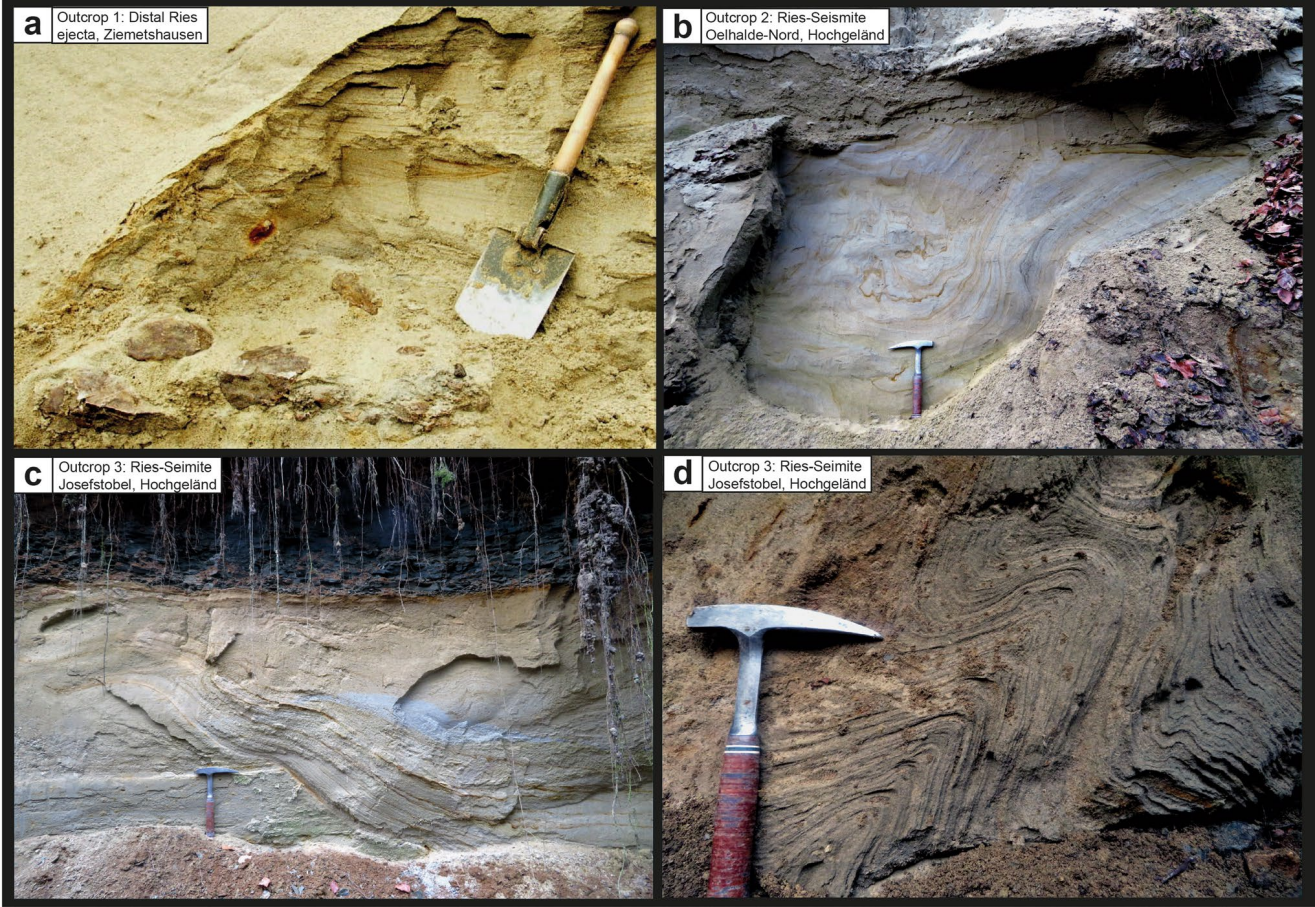
Photographs of the most impressive details of the Ries seismite and distal Ries ejecta that caps the Ries seismite from the outcrops discussed in the present study. (**a**) Primary (in situ deposited) horizon of distal Ries ejecta with angular Upper Jurassic limestone components from the former sandpit Ziemetshausen (photographs: **a** was taken by Peter Bockstaller, Schopfheim, in 2005; **b**–**d** taken by V.J.S. in 2020–2021, spade and hammer for scale). (**b**) Slump with internal convolute bedding in the Ries seismite from the Tobel Oelhalde-Nord (Hochgeländ); (**c**) Thrust fault-like slump in the Ries seismite from the Josefstobel (Hochgeländ plateau south of Biberach an der Riß); (**d**) Distinct fault- and fold-like structures in convolute bedding in the lower (Ries) seismite horizon from the Josefstobel (Hochgeländ plateau south of Biberach an der Riß).

**Figure 7:**
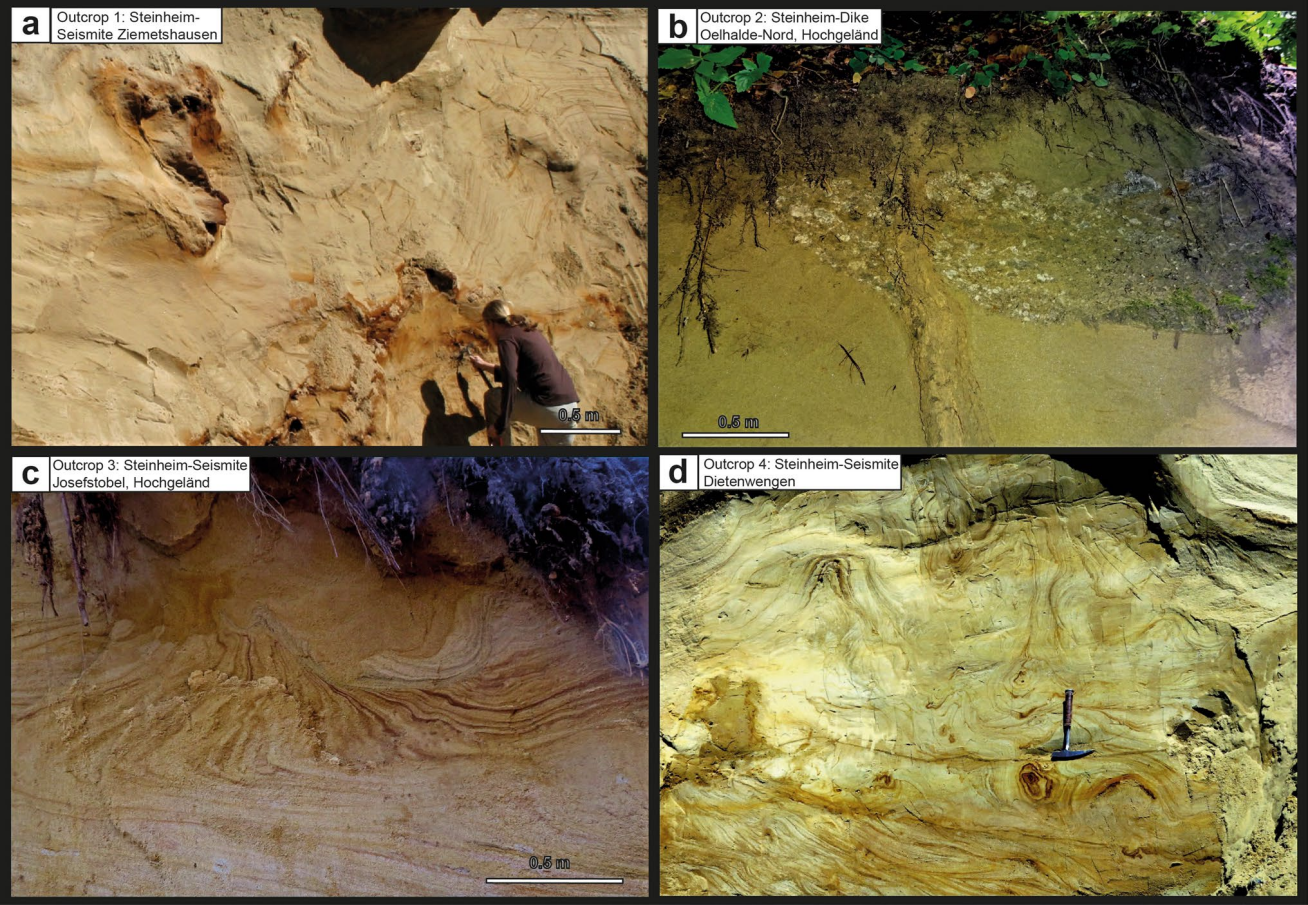
Photographs of the details of the Steinheim seismite and clastic dikes from the four outcrops discussed in the present study; (**a**) The 4–5 m-thick Steinheim seismite in the Ziemetshausen sand pit with slumps in the upper parts and convolute bedding in the lower parts of the seismite. A reworked (secondary) layer of distal Ries ejecta is integrated into folding of convolute bedding and clearly demonstrates that the upper seismite horizon is of post-Ries age; (**b**) Giant clastic dike cross-cutting a horizon of reworked OSM material approximately 2–3 m above the Ries seismite and distal Ries ejecta in the Tobel Oelhalde-Nord (Hochgeländ); (**c**) Slump in the Steinheim seismite from the Josefstobel (Hochgeländ plateau south of Biberach an der Riß); (**d**) Ball-and-pillow structures in the upper (Steinheim) seismite horizon at the Dietenwengen sand and gravel pit, hammer for scale. Photograph (**a**) was taken by E.B. in 2008; photographs: (**c**)and (**d**) were taken by V.J.S. in 2021 (**b** in 2019).

In the former, no longer accessible sand pit near Ziemetshausen^36^ (48°17′17.5″ N/10°30′46.5″ E, 508–529 m.a.s.l.; Fig. 2), the Ries seismite horizon is presumably present, but largely buried. A conspicuous horizon of primary, in situ-deposited distal Ries ejecta was observed by a member of our working group near the base of the sand pit (E.B.) in 2008 and by a colleague (pers.com. P. Bockstaller) in 2005 (Fig. 2, 6a). Only a few tens of centimetres of OSM sands with convolute bedding were visible on an outcrop scale at the basal minig level between 2005 and 2008, suggesting this outcrop also contains the several metres thick Ries seismite underneath the ejecta horizon, in analogy to exposures elsewhere in the NAFB^3,16,27^. The Ries ejecta horizon is overlain by a minimum of 12 m of undisturbed, cross-bedded OSM sands. Secondary (reworked) distal Ries ejecta occur within this succession as individual redeposited limestone components or reworking horizons. One of the limestone clasts shows internal shatter cones^15^ (Fig. 2). A second, at least 4 m-thick, upper seismite horizon follows that shows large slump structures over a lateral extension of more than ten metres (Figs. 2, 6a), as well as distinct outcrop-scale faults. It is noteworthy that a horizon with reworked distal Ries ejecta is also included in the fold-like deformation tracing a large slump within the upper seismite horizon (Fig. 7a). The upper seismite is topped by two metres of undisturbed, cross-bedded OSM sands and another ~ 1 m of Quaternary deposits and soil.

### Outcrop no. 2: Tobel Oelhalde-Nord

In the Tobel Oelhalde-Nord (48° 02′24.9″ N, 09° 49′52.0″ E, 504–525 m.a.s.l.; Fig. 3), the uppermost part (~ 3 m) of the Ries seismite is exposed near the base of the ravine. This outcrop was already described in parts in previous studies^3,16^. The seismite is represented by metre-sized slumps (Figs. 3, 6b) and convolute bedding and is overlain by a primary horizon of in situ-deposited distal Ries ejecta with a portion of limestone components showing distinct shatter cones (Fig. 3). The overlying ~ 15 m of cross-bedded OSM sands are basically undisturbed. However, a giant, at least ~ 17 m-tall, clastic dike (Figs. 3, 7b) cross-cuts the lower seismite, the distal Ries ejecta horizon, and the otherwise undisturbed OSM sands, ending in a small fossil sand volcano (Fig. 3)^16^. This sand volcano, i.e., the site of local sand extrusion, presumably marks the paleo-land surface at the time of the seismotectonic event that created the dike. A laterally extensive upper seismite horizon sems to be absent in this outcrop, which may suggest a rather dry state of the surface-near sandy deposits at the time of the seismic event. However, at least 13 m of undisturbed Molasse deposits that separate the two seismite units (i.e., the Ries seismite horizon/distal Ries ejecta and the cross-cutting giant clastic dike at the extrusive sand volcano level)^16^ provide tangible sedimentologic evidence for two temporally independent seismotectonic events in the Tobel Oelhalde-Nord exposure.

### Outcrop no. 3: Josefstobel

In the Josefstobel (48°01′61.2″ N/9°82′69.8″ E, 593–633 m.a.s.l.; Fig. 4), the at least 10 m-thick lower (older) seismite horizon includes sand spikes (locally known as ‘Zapfensande’) that were recently interpreted as a new, high-energy form of seismite^27^, associated with OSM sands showing distinct convolute bedding (Fig. 4) and thrust fault-like slumps (Figs. 4, 6c,d). In this particular outcrop, distal Ries ejecta that commonly cap the Ries seismite^3,16^ are absent and substituted by a 1 m-thick layer of dark, grey fluvio-lacustrine claystone that seems to have formed within a Middle Miocene local pond or lake. This clay layer rests on top of the Ries seismite with sharp contact and is partially rich in fossil leaf imprints of cinnamon trees (*Daphnogene polymorpha*). The claystone unit is overlain by about 14 m of undisturbed, cross-bedded OSM deposits, which are in turn capped by a ~ 2 m-thick second seismite horizon (Figs. 4, 7c). That upper seismite layer is, similar to the Ries-seismite 15 m below, also characterized by slumps and convolute bedding. In addition, clastic dikes occur in the otherwise undisturbed portion of OSM deposits sandwiched by the two seismites. The upper seismite horizon is, moreover, partially overlain by a bone bed with fossil remnants of vertebrates including turtles and tortoises (*Trionyx, Testudo*) and the Miocene deer species *Dicrocerus elegans*^37^. Fossils of this cervid with forked antlers are restricted to the European Land Mammal Zones (ELMZ) MN 5 and MN 6 but become increasingly abundant in MN 6 (evolution level Sansan). The findings of *Dicrocerus* in a relative high lithostratigraphic position indicate the upper seismite horizon in this outcrop can be biostratigraphically placed most likely in the MN6 biozone. Finally, the seismite unit is overlain by several metres of undisturbed OSM sands topped by Quaternary deposits and soil.

### Outcrop no. 4: Dietenwengen

In the Dietenwengen sand and gravel pit near the small town of Eberhardzell (Fig. 5; 48°02′17.6″ N/9°86′45.8″ E, 619–640 m.a.s.l.), the lower, Ries-related seismite horizon is not exposed. The lower seismite horizon and/or distal Ries ejecta are probably buried under at least ~ 5–15 m of OSM deposits. Nevertheless, the ~ 0.5–2 m-thick upper seismite unit (Figs. 5, 7d) is well-exposed and characterized by distorted and partially dewatered OSM deposits overlain by distinct ball-and-pillow structures, intensive convolute bedding with flame structures (Figs. 5), and cross-cut by clastic sills and dikes. Here, the upper seismite horizon is often directly underlain or replaced by 3–4 m (depending on the mining situation) of cross-bedded sands with several internal layers that contain reworked OSM deposits with larger clasts and fossils of plants, molluscs, reptiles and mammals. The upper seismite horizon is followed by a ~ 14 m-thick sequence of undisturbed, cross-bedded sands, also cross-cut by clastic dikes and sills, as well as faults along which variable offset of the Molasse deposits can be observed. These yellowish sands (‘gelbe Molasse’) are, in turn, followed by a few metres of grey OSM deposits (‘graue Molasse’) that form the upper end of the sand and gravel pit. The thin subvertical dikes and wedges in the cross-bedded sands on top of the upper seismite horizon, as well as brittle deformation features, may have been caused either by the overburden of glaciers during the Pleistocene, local and minor tectonic activity, or may have formed simultaneously with the upper seismite horizon.

In 2021–2022, various fossils were discovered at the Dietenwengen sand pit by one of our group (V.J.S.), for instance invertebrates including gastropods (*Cepaea*) and bivalves (*Margaritifera, Unio*), lower vertebrates like turtles and tortoises (*Trionyx, Mauremys, Testudo, Titanochelon*) and crocodiles (*Diplocynodon*), and bones and teeth of at least 6 taxa of mammals. The mammal fossils occur in bone beds underlying (or laterally replacing) the upper seismite and include teeth and bones from *Steneofiber* sp.*, Dorcatherium* cf. *naui, Brachypotherium brachypus*, well preserved antler fragments and teeth of the cervid *Dicrocerus elegans*^37^ and a fragment of a large molar of the proboscidean (Tertiary elephant) *Gomphotherium* cf. *steinheimense*^38,39^. Furthermore, we report a single, but well preserved carnivore tooth (M 1 inf.), which fits morphometrically very well with the rare taxon *Alopecocyon* cf. *goeriachensis* (TOULA), an extinct relative of the small red panda (fam. Ailuridae) that today inhabits the Himalayas and SW China. Fossil remnants of the carnivor genus *Alopecocyon* are only known from very few localities in mid-Miocene deposits (e.g. Sansan, Göriach) in Europe and Pakistan that span the ELMZ MN 6 and MN 7 biozones. The faunistic and biostratigraphical element of *Alopecocyon* is, hence, crucial for dating the upper seismite horizon at the Dietenwengen locality.

### Outcrops with clastic dikes

Smaller and larger (and at least one giant) clastic dikes and sills occur in several outcrops in the Hochgeländ plateau south of Biberach an der Riß, for instance in the ravines Tobel Oelhalde-Nord and Oelhalde-Süd^3,16^ (Fig. 3, 7b), Josefstobel (this work; Fig. 4), and in the Dietenwengen sand pit (this work; Figs. 5, 7d). We also found clastic dikes and sills in ravines in the Ravensburg area (e.g., at the Kleintobel) and at Untereichen south of Ulm^27^. In total, more than 30 clastic dikes, including dike swarms, have thus far been recognised in the Hochgeländ plateau. Most dikes and sills start below and cross-cut the Ries seismite and the ejecta horizon and, therewith, clearly postdate the latter. Other clastic dikes seem to originate somewhere between the lower (Ries) and the upper seismite horizon. We also found some rare small dikes and sills that cross-cut the upper seismite horizon in the Dietenwengen sand pit. Some dike- and wedge-like features run from top to bottom and are presumably of Pleistocene age. Additional small dikes and sills start within or slightly below the upper seismite horizon, and their formation can be interpreted as ‘co-seismite’ intrusion more or less simultaneous to the formation of clastic dikes and convolute bedding^40^. Hence, we argue the dikes/sills and the convolute bedding in the upper seismite horizon are of mid-Miocene age and the result of the same seismic event.

## Discussion

### The Ries seismite: Evidence for long-distance seismic shaking

The Ries seismite, i.e., the ~ 10–15 m-thick lower seismite unit and overlying distal Ries ejecta^3,14,18^, forms a unique continental seismite-ejecta couplet within a distance of 180 km from the crater^3^. The structural inventory within the Ries seismite, characterized by distinct soft-sediment deformation and dewatering structures described recently (such as sand spikes^3,27^) indicates far-reaching seismic effects of the 14.81 Ma Ries impact^5,6^. The seismite, which is exposed at several localities within the NAFB^3,16,27^ features metre-sized slumps, usually with NW–SE-striking slump axes, convolute bedding, and ball-and-pillow and flame structures^3^. The dip of the slumps and the strike of slump axes are both consistent with a seismic source in the Ries crater region^3,27^. Such soft-sediment deformation features in continental deposits are typical of seismites produced by major earthquakes^41,42^. The horizon of distal Ries ejecta^15,16^ that caps the seismite unit (outcrops nos. 1 and 2^3,16,27^) provides direct evidence that the Ries impact was the trigger of the seismic event that, in turn, caused soft-sediment deformation within a radial distance of at least ~ 100–180 km from the impact site^3,17,18^. The energy imparted into the target deposits by the impact of distal impact ejecta components is much too low to induce seismic shaking of metre-thick (up to 15 m) Molasse deposits^16,19^. A recent study^31^ suggests the Ries impact-earthquake even reached northern Switzerland and produced seismic sand diapirs and sand volcanoes in surface-near OSM sediments near Winterthur, more than 200 km away from the Ries impact structure. The restricted occurrence of the Ries seismite within the NAFB suggests the seismically-induced production of soft-sediment deformation required specific lithophysical properties of the near-surface Molasse sediments, such as (locally) water-saturated unconsolidated sands in combination with underlying waterlogging strata^13,16,27^.

The newly described Ries seismite occurrence (outcrops nos. 1 and 3) expand the known area of distribution of the Ries seismite and the associated primary horizon of distal Ries ejecta (outcrops no. 1 and 2 and additional sites already described^3^). In all four exposures, bedding conditions and features caused by soft-sediment deformation are remarkably similar. The overlying Ries ejecta layer is either developed as a primary, in situ-deposited horizon (outcrops nos. 1 and 2) or as a secondary, reworked layer of ballistically transported and shocked clasts of Upper Jurassic limestone (outcrop no. 1). In some of the outcrops described earlier^3^, the overlying layer does not display unequivocal rock components ejected from the Ries crater, but consists of coarse-grained, locally reworked pebbles, cobbles, and boulders. In outcrop no. 3, the ejecta layer is substituted by dark grey to black claystones, which we interpret as fluvio-lacustrine fines deposited in a pond or small lake dammed by slumped OSM deposits immediately after the Ries impact. We did not find any coarse-grained components ejected from the Ries crater within these deposits, which suggests ballistically transported distal Ries ejecta was deposited in patches and not as a continuous ejecta blanket. The findings from the four outcrop localities combined provide a detailed sedimentologic and temporal account of the profound environmental impact the Ries event had on the surrounding paleo-landscape^3^ (this study).

### Is the upper seismite linked to the Steinheim impact?

The upper seismite horizon in the newly discovered outcrops, separated from the deeper Ries seismite by a ~ 10 to 15 m-thick succession of essentially undisturbed OSM sediments, resembles the Ries seismite in many ways, although the entire unit is usually much thinner and only attains a thickness of ~ 0.5 to 2 m (Hochgeländ area and Dietenwengen, ~ 70 km south of the Steinheim basin; outcrops nos. 2, 3, and 4; Figs. 2, 4 and 5). At Ziemetshausen, some ~ 50 km from Steinheim, the seismite unit appears to reach a greater thickness of ~ 4–5 m (outcrop no. 1; Fig. 2). The most common features caused by dewatering are convolute bedding, ball-and-pillow and flame structures in the lower parts, and metre-sized slumps with internal convolute bedding in the upper parts of the seismite horizon. The thinning of the upper seismite horizon (from 4–5 m at a distance of ~ 50 km to 0.5 to 2 m at a distance of ~ 70 km) correlates with increasing distance from the Steinheim impact cater region, which may suggest a genetic link between the upper seismite and the Steinheim impact event as a likely trigger mechanism. In turn, thinning of this seismite unit towards the south is at odds with a seismic source in the Alpine region^3,16^.

Both the lower (Ries) and the upper (presumably Steinheim) seismite horizons are overlain by several metres of undisturbed OSM deposits in each of the outcrops (nos. 1–4) studied (Figs. 2, 3, 4 and 5), and accordingly, both seismite horizons were deposited within the ‘Fluviatile Untere Serie’ unit of the OSM^12,34,35,43^. The stratigraphic position of the upper seismite near the top of the ‘Fluviatile Untere Serie’ suggests this seismite was deposited around ~ 14.3 Ma or somewhat later, i.e., at least ~ 0.5 Myr after the 14.81 Ma Ries impact. The (lower) Ries seismite horizon is often capped by a primary or reworked horizon of distal Ries ejecta, or by contemporaneous continental flash-flood deposits. The upper seismite horizon is locally overlain by reworked clastic material (compare to outcrop no. 3, Fig. 4), suggesting a contemporaneous continental flash-flood event that followed the second seismic event. However, none of the material overlying the upper seismite horizon has, thus far, shown evidence of an origin as distal ejecta derived from the Steinheim impact (e.g., small shatter cones or shocked mineral grains). At a distance of ~ 50–70 km from the Steinheim crater (at Ziemetshausen and in the Hochgeländ area and Dietenwengen), the existence of distal Steinheim ejecta is not to be expected or, if present, the layer would likely be less than one millimeter thick and, therefore, barely identifiable within the surrounding sands^19,20^. According to numerical simulations of ejecta emplacement, the maximum grain size of distal Steinheim ejecta within this distance would be less than one cm^26^. In contrast to the much larger Ries, the continuous ejecta blanket of the ~ 4 km-diameter Steinheim crater would only have reached a radial distance of 8–12 km from the crater rim^22^. The most distal Steinheim ejecta of considerable grain size would have landed some ~ 30 km from the source crater. In addition, it is unlikely to find shocked mineral grains in the thin, distal portion of any potential Steinheim ejecta layer. Even within the impact breccia that fills the Steinheim crater, mainly composed of brecciated Middle and Upper Jurassic limestones and sandstones, shocked quartz seems to be extremely rare, if present^9^. Therefore, the search for shocked quartz or zircon in deposits that cap the Steinheim seismite, which would establish a firm link between the seismite and an impact-earthquake, is not promising. A systematic search for possible traces of a fine-grained Steinheim-produced ejecta layer (and/or ablation products from the Steinheim projectile), if ever deposited and preserved^22,44^, is a future endeavour.

### Biostratigraphic arguments for a Steinheim-age upper seismite

Independent support for a Steinheim-related impact-earthquake comes from fossils found associated with the upper seismite. Whereas the distal ejecta layer that rests on top of the Ries-produced seismite contains fossils of a mammal fauna characteristic of the transition from ELMZ MN 5/6 to lower MN 6 within the earlier Langhian^3,14,15,35,45^, the mammal remains (*Alopecocyon* cf. *goeriachensis, Dicrocerus elegans; Gomphotherium* cf. *steinheimense;*) found directly below, within, or on top of the upper seismite are together indicative of ELMZ MN 6 to the transition zone MN 6/7. In particular, the occurrence of the genus *Alopecocyon* and the comparatively large amount of fossil antler remains of *D. elegans* associated with the upper seismite exposed at Dietenwengen sand pit and Josefstobel, in combination with a fossil tooth of an evolved, relatively large, proboscidean *Gomphotherium* cf. *steinheimense*, suggests these Mid-Miocene deposits are similar in age to those known from Sansan (France), the international type locality of the ELMZ biozone MN 6^46,47^ or slightly younger, i.e., MN 6 to MN 6/7^48^. These biostratigraphic constraints are in agreement with the immigration of *Dicrocerus* deer into Europe from Asia and its first appearance in mammal biozone MN 5, followed by its typical widespread occurrence in biozone MN 6^35,36,49^, and the size evolution of *Gomphotherium*, with larger individuals being prevalent later during the Neogene^50,51^. Overall, the upper seismite seems to indicate an age corresponding to biozone MN 6 or the transition zone MN 6/7. In particular, the relatively thick and very distinct upper seismite horizon detected at the Dietenwengen sand pit is underlain by bone beds that offer a rich fauna indicative for the transitional ELMZ zone of MN 6 to MN 6/7.

Two independent aspects of biostratigraphic correlation regarding the two separate seismites are of particular relevance. First, both the lower (Ries) and upper (Steinheim?) seismite horizons are separated by one entire ELMZ biozone—essentially the interval between the MN 5/6 and MN 6/7 transition zones. Second, both seismites can be biostratigraphically correlated with the basal crater lake sediments at the Middle Miocene Ries and Steinheim impact craters, respectively. Since the Ries impact produced a distinct ejecta marker horizon^3,14,15^, that stratigraphic correlation is further corroborated in the field south of the Ries crater. The Ries ejecta horizon occurs within the MN 5/6 transition zone, i.e., at or near the base of biozone MN 6^3,14,27,35^. These constraints are consistent with the MN 6 mammal and avian fauna at the Ries lake^33,52^, a rather hostile, evaporitic soda lake^53,54^ that evolved within the newly formed impact crater.

The upper seismite horizon corresponding to the higher MN 6 biozone and probably the transitional interval MN 6/7 correlates very well with the (basal) Steinheim crater lake deposits^1^. Although Steinheim served as the type locality for the European mammal biozone MN 7^55^, the initial deposition of lake sediments at Steinheim may have started in late MN 6 or in MN 6/7 transition time (during the local *Gyraulus kleini*, *G. steinheimensis*, and *G. sulcatus* periods^10^); however, from a biostratigraphic point of view, the presence of MN 6 in the Steinheim crater lake is not finally proven. Hence, there seems to be good biostratigraphic correlation between the deposition of sediments within the newly formed Steinheim crater lake^1,12,56^ and the emplacement of the upper seismite horizon in the NAFB as constrained by its fossil content.

**Figure 8:**
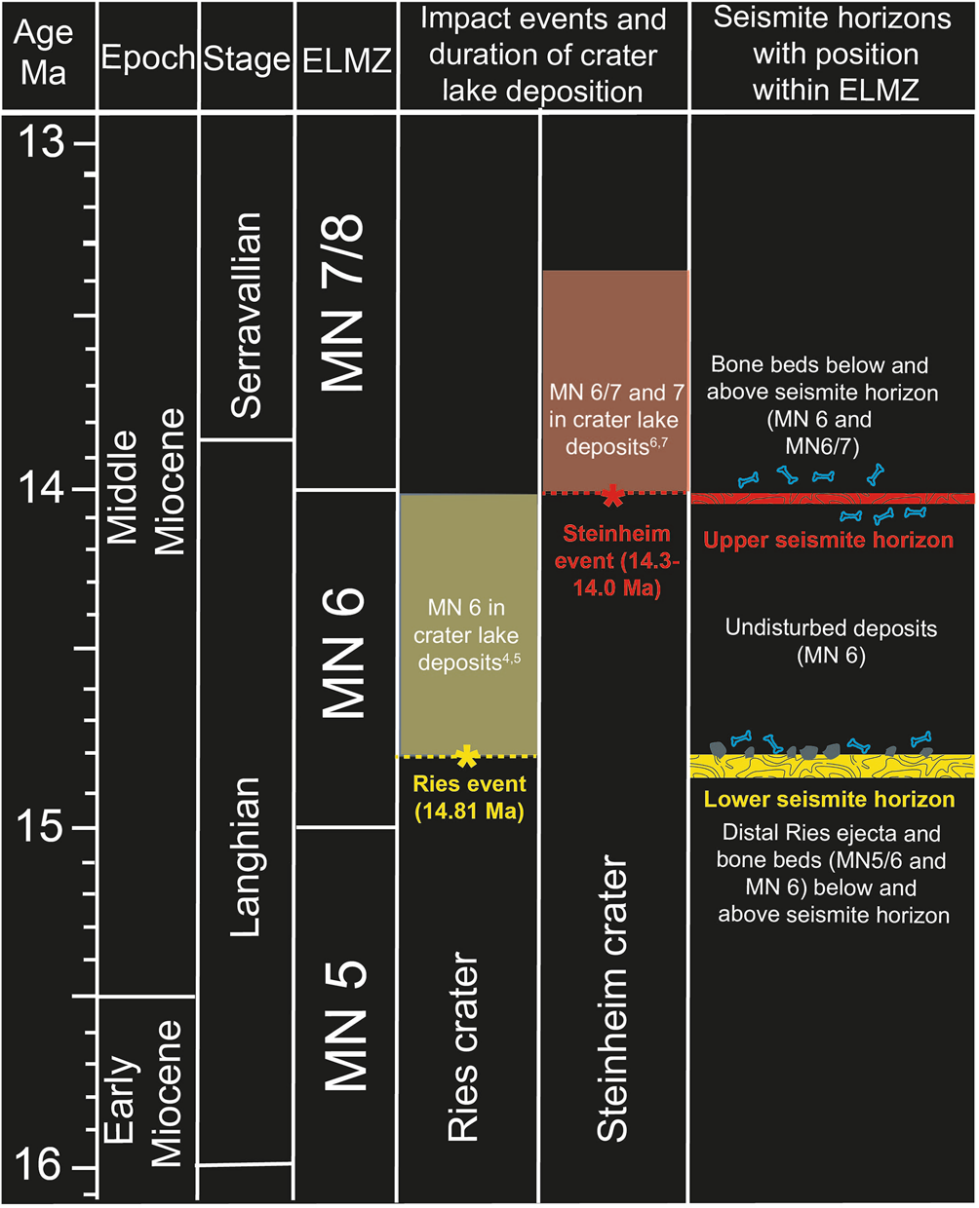
Chrono-^59^ and biostratigraphic^12^ classification of the crater infill of the Ries and the Steinheim impact structures and the lower and upper seismite horizons. The lower seismite horizon of Ries age^5,6^ is embedded in OSM deposits that contain faunal elements characteristic for the ELMZ MN 5/6 to MN 6. The lowermost Ries crater lake deposits are dominated by the same faunal assemblage. The upper seismite horizon is under- and overlain by faunal elements of the ELMZ MN 6 to MN 6/7, strikingly consistent with identical faunal elements that occur in the lowermost Steinheim crater lake deposits; *ELMZ* European Land Mammal Zone.

In summary, there seems to be a clear time gap between the formation of the Ries seismite (approximately MN 5/6 transition) and the upper seismite presumably related to the Steinheim impact (upper MN 6 or transition MN 6/7), which is apparent based on biostratigraphic evidence (Fig. 8). That time gap is further evidenced by a 10 to 15 m-thick layer of undisturbed sands that separates the two seismite layers in the field (compare Figs. 2, 3, 4 and 5). The time difference between the formation of the two seismites may help solve a long-held geologic problem that originated from the alleged synchronicity of the Ries and Steinheim impacts^13,33^. According to that model, the Ries crater lake formed soon after the impact (MN 5/6) in the mid-Langhian^12^, following the impact at 14.81 Ma^5,6^. In contrast, following that theory, the Steinheim crater would have stayed ‘dry’ for an extended period of time (several kyr) before the crater depression was filled with water and lake deposits in MN 7 time^12,33^.

The new biostratigraphic impact crater-seismite correlation presented in this study reconciles previously conflicting theories regarding the timing of impact crater formation as it now allows for a temporal gap between the Ries and Steinheim impact events. Presumably, both craters initially represented morphological depressions without much fluvial drainage that would have filled up with water soon after impact, as expected in a deeply water-saturated paleolandscape in the Middle Miocene^57^. In fact, the pervasive alteration of impact melt particles within the Steinheim impact breccia^9^ and the occurrence of thermal spring mound deposits on top of the central uplift (exemplified by the aragonitic ‘Wäldlesfels’^1^) demonstrate that the Steinheim Basin hosted a sizeable (but rather short-lived) post-impact hydrothermal system and was almost completely filled with water. During that phase, the prominent central uplift of the Steinheim crater was, at least temporarily, partially to entirely submerged. While the Ries impact has been Ar–Ar-dated at a precise age of ~ 14.81 Ma^5,6^, the current best-estimate age for the Steinheim impact is derived entirely from biostratigraphic constraints. Assigning a late MN 6 to transitional MN 6/7 age to the impact, the Steinheim event most likely occurred in the terminal Langhian^12^ or earliest Serravallian^58^, around ~ 14.3 to 14.0 Ma. Considering the most recent high-precision geochronologigic results for the Langhian/Serravallian boundary (13.82 Ma^59^; ~ 13.8 Ma^60^), a late Langhian Steinheim impact scenario appears most likely. Overall, a time interval of at least ~ 0.5 Myr (and potentially as long as ~ 0.8 Myr) seems to separate the Ries and Steinheim impact events despite their geographic proximity. It, however, remains unclear at this point whether, and to what extent, the two impacts are causally connected to the distinct faunal turnover in European land mammals marked by the MN 5/6 (within uncertainty Ries-age) and MN 6/7 (approximately Steinheim-age) biozone transitions^35^ (see also discussion therein).

### Significance of clastic dikes

In addition to the lower and upper seismite units described above, outcrop-scale clastic dikes first described near Biberach an der Riß (along the flanks of the Tobel Oelhalde-Nord) and Ravensburg (Kleintobel^16^) are eye-catching features in the local outcrops, structurally linked to paleoseismic activity. Additional clastic dikes and dike swarms occur in a number of exposures along ravines in the Hochgeländ plateau, for instance the Josefstobel, but also in the Untereichen sand and clay pit south of Ulm^27,34,43^ (Fig. 1). The clastic dikes and dike swarms are earthquake-produced structures^41^. In terms of their stratigraphic setting and age, they commonly cross-cut the Ries seismite and ejecta layer where present, and also cut through the sequence of undisturbed post-Ries deposits that lie between the lower and the upper seismite. Hence, the clastic dikes clearly postdate the Ries impact event and earthquake^3^. The largest known clastic dike reported from the Tobel Oelhalde-Nord was, therefore, tentatively linked to the Steinheim impact event that presumably postdates the Ries by a few kyr^16^. It is noteworthy that none of the clastic dikes that cross-cut the Ries seismite/distal ejecta-couplet also cross-cut the upper seismite horizon. This suggests the clastic dikes and the upper seismite, produced by a second large Middle Miocene earthquake, are potentially genetically linked. In other words, the paleoearthquake that produced the upper seismite potentially also created the clastic dikes.

As discussed previously, Alpine seismic sources and/or tectonic activity associated with major fault and graben zones within the South German Block (e.g., the extensional Upper Rhine graben or the Hohenzollern-/Lauchertgraben)^61^ are unlikely the trigger mechanism for the clastic dikes, which occur far away from any major endogenic tectonic structure^16^ and also postdate the main phase of Alpine tectonism by a few Myr^3,27,62^. Likewise, Mid-Miocene seismo-volcanic activity within the Hegau^63^ or the phreatomagmatic Urach-Kirchheim volcanic fields^64^ are presumably too far away from the seismite occurrences and too weak of a seismic source to be considered viable trigger mechanisms for the widespread formation of soft-sediment seismites in large areas of the NAFB^16^ (see discussion therein). Therefore, the most likely scenario is that the palaeoearthquake responsible for the formation of the upper seismite and the clastic dikes was triggered by the Steinheim impact event, only some ~ 50 to 100 km away, with a calculated moment magnitude of around M_w_ 7 (depending on the effective diameter of the Steinheim Basin before erosion, within a range between 4 and possibly 5 km^26^). In contrast to the Ries impact-earthquake, which mainly produced a seismite unit suggestive of a water-saturated paleoenvironment (i.e., mainly soft-sediment deformation, such as convolute bedding, flame and ball-and-pillow structures; as well as sand spikes^3,27^), the Steinheim earthquake seems to have created a variety of palaeoseismic deformation features in the host sediment, including structures similar to those in the Ries seismite, but also subvertical clastic dikes that indicate a dryer state of the local bedrock substrate^16^. As an exemplary case for a relatively dry bedrock paleosurface, no distinct upper seismite horizon developed in the outcrop Tobel Oelhalde-Nord (Hochgeländ), but instead a giant clastic dike cuts through the Ries seismite and the overlying OSM deposits, reaching the former paleo-land surface by forming an extrusive sand volcano^16^. The difference of physical substrate properties is plausible when one takes into account the climatic change from warmer, humid conditions at the time of the Ries impact (14.81 Ma) towards a cooler and dryer climate only a few kyr later, when the Steinheim event occurred (~ 14.3 to 14.0 Ma). The time interval between 15 and 14 Ma, in particular, indicates a major decrease in reconstructed clumped isotope temperatures (from NAFB paleosoils) following the ~ 14.9 Ma Miocene Climate Optimum^65,66^. That change in climate led to a stronger seasonality and less humid conditions in Central Europe. At the time of the Ries impact, the palaeolandscape was characterized by a high groundwater level that covered deeper parts of the Molasse deposits up to the palaeosurface. Those water-saturated conditions allowed the Ries-impact earthquake to cause pervasive stirring of soft, seismically fluidized sediments. In contrast, the earthquake induced by the Steinheim impact-earthquake seemingly only affected the uppermost portions of the water-bearing sediment, causing widespread but limited dewatering that produced a seismite horizon of ~ 4–5 m in thickness in Ziemetshausen and only up to ~ 2 m in the Hochgeländ. Instead, the Steinheim earthquake also produced clastic dikes in the deeper parts of the Molasse deposits, where isolated pockets or layers of water-saturated sand occurred on top of thick clay or marl layers that served as local aquitards in an otherwise rather dry bedrock with a deep groundwater level^16^.

### Alternative trigger mechanisms for the production of widespread seismites in the NAFB?

**Figure 9:**
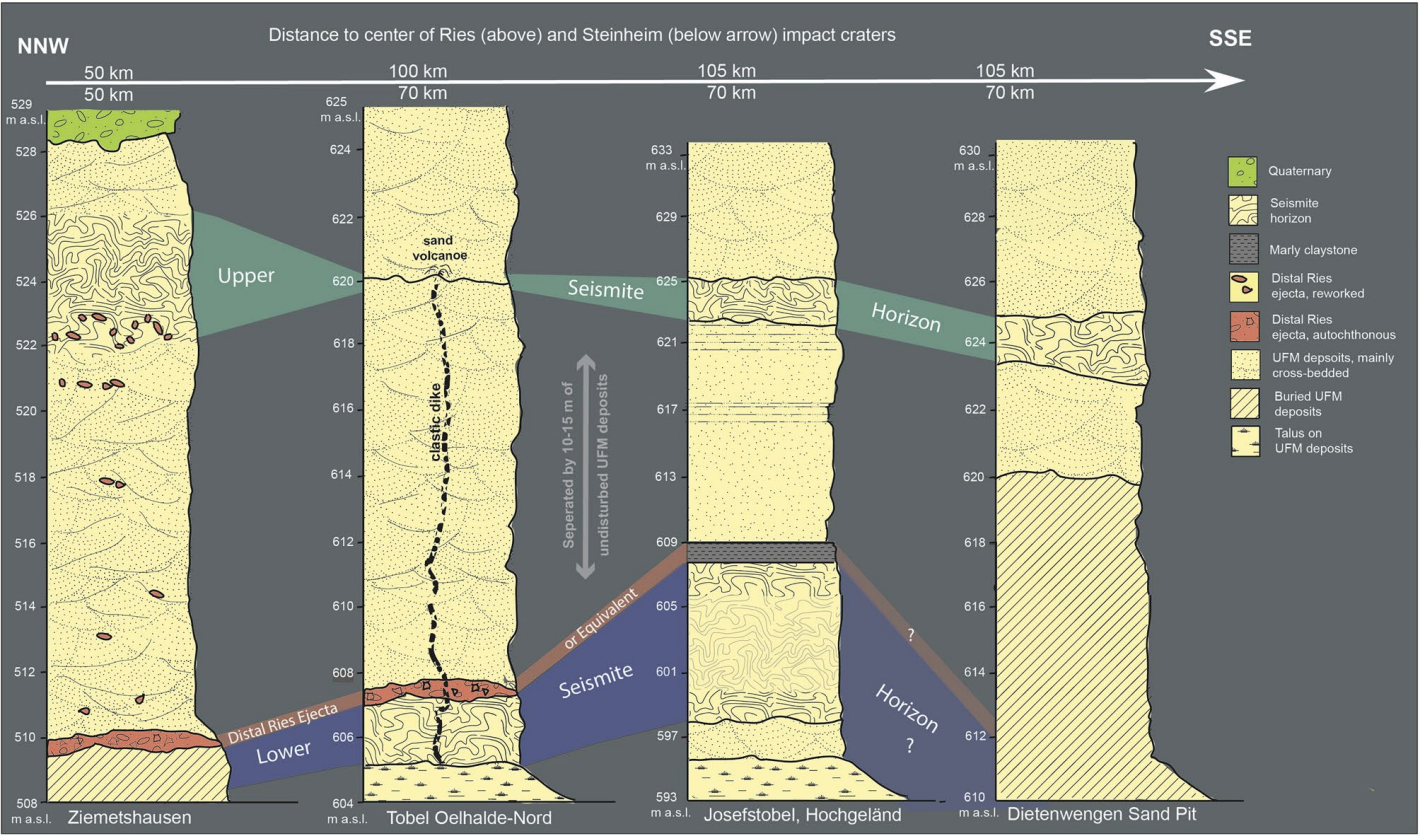
Simplified lithostratigraphic columns illustrating the four outcrops described and discussed in the present study. In these outcrops, a lower Ries seismite horizon capped with distal Ries ejecta (or equivalent) and an upper Steinheim seismite horizon (or equivalent) are clearly associated by appearance and altitude above the sea-level. Note that the outcrop Ziemetshausen with significant divergent altitude is situated ~ 50 km north of the other three outcrops.

A number of potential trigger mechanisms and source regions should be taken into account to explain the formation of the seismites in the NAFB. While the lower seismite unit is unequivocally linked to the Ries impact through its cap of impact ejecta, various geologic processes may have produced the upper seismite (Fig. 9), from which no overlying ejecta have, thus far, been reported (see also discussion above and why it is unlikely or even impossible to find distal Steinheim impact ejecta that far away from the source crater).

**Figure 10:**
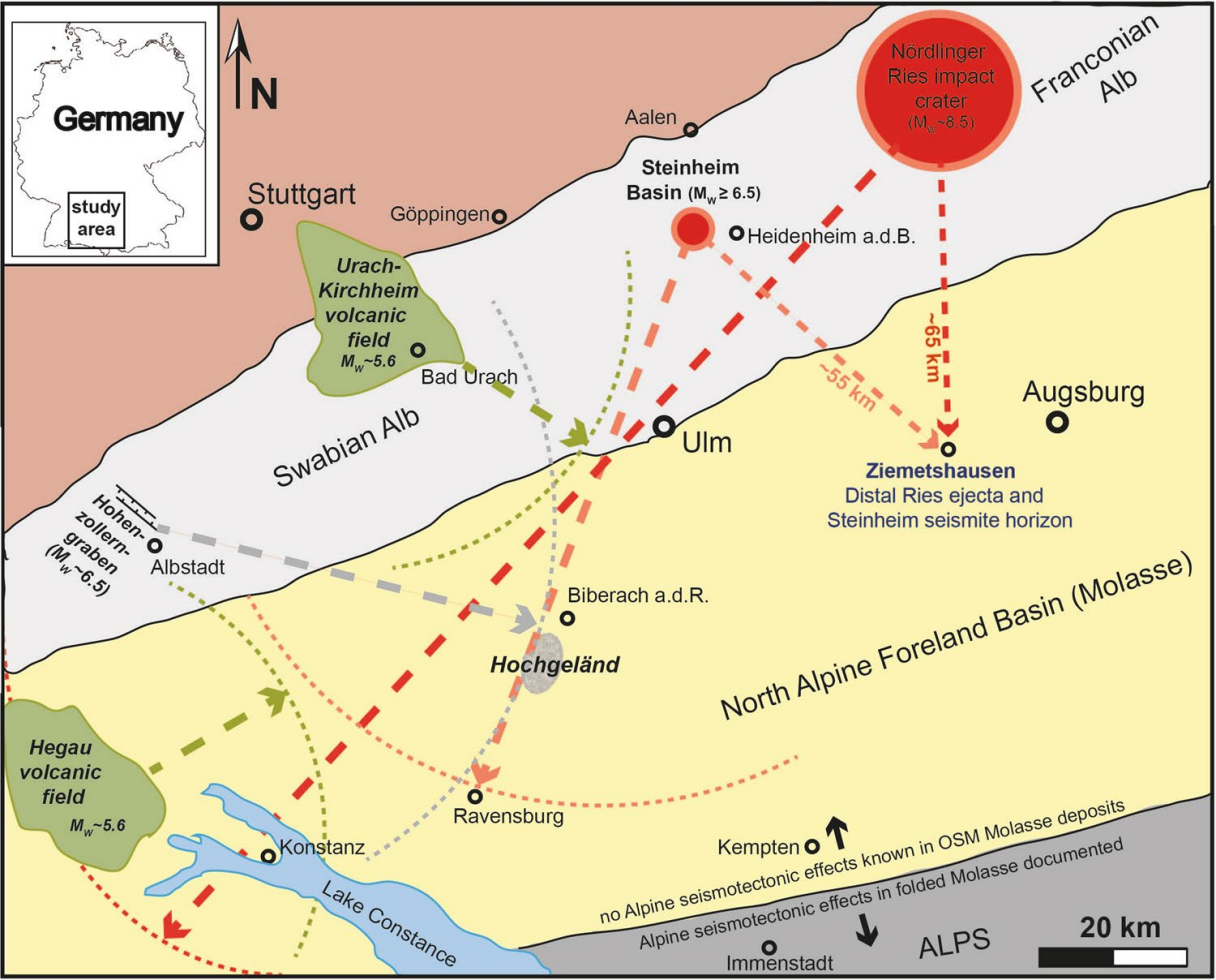
Geographic and geologic situation in the study area in southern Germany, northern Switzerland and Austria. All seismic sources active in the study area in mid-Miocene times are depicted including their estimated potential of maximum earthquake intensity in moment magnitude (M_W_) (see^16^ for conversion of magnitudes in Richter scale M_L_ to M_W_; and^3^, their Supplementary Table 2) and the maximum distance of the potential formation of earthquake-induced seismites in affected sediments within the circle segments^16^. The dashed circular lines mark, hence, the limit of potential seismite formation at a magnitude of ~ MW < 5^41,42^ for each known seismic source acitive in Middle Miocene. The large red arrow shows the maximum distribution of seismites evidently induced by the Ries impact-earthquake within 205 km from the crater; the large orange arrow marks the distribution of seismites attributed to the Steinheim impact-earthquake^3,16^; green arrows show the estimated influence sphere of potential volcanic-induced earthquakes, and the grey arrow shows the potential maximum influence of the seismotectonic feature of the Hohenzollerngraben. The seismotectonic source of the Upper Rhine Graben with its distance of more than 150 km to the Hochgeländ area and more than 200 km to the Ziemetshausen outcrop is not considered, because seismite formation at such a distance is very unlikely. Note that no other seismic source beside the Ries and Steinheim impact events had the potential seismic capacity to impart enough energy into the Ziemetshausen sedimentary target, sufficent to form metre-thick seismite horizons.

With respect to volcanically induced seismicity, the seismic potential of the Hegau and Urach-Kirchheim volcanic fields, potentially causing earthquakes of M_W_ ~ 5 to 6^63,64^, is presumably too low to be considered a convincing trigger mechanism for widespread, large-volume seismite formation across the NAFB. Seismites induced by major Alpine tectonic acitivity^62,67^, which predates both the Ries and Steinheim impact events and, hence, did not affect the unfolded Upper Freshwater Molasse^34^, are known mainly in older (marine) Molasse deposits in Switzerland and southern Germany, but are limited to the area south of the line Lake Constance—Oberstaufen—Immenstadt marked in Figs. 1 and 10^3,16,62,67^ (see also extensive discussion in^3,16^) .

In Fig. 10, we illustrate how the Biberach and the Ravensburg areas might have been influenced by mid-Miocene seismic events along the Upper Rhine Graben or in the Hohenzollerngraben domain in SW Germany. The estimated maximum earthquake strength along these prominent tectonic features may have reached magnitudes M_W_ 6 to 6.5^16,61^, also taking into account the maximum distance of seismite formation based on the systematic study of strong historic earthquakes^16,68^ (see Table 1 in^16^ and extensive discussion therein; see also^3^, their Supplemetary Table 1 and “Methods”).

The distance of ~ 150 km between the large tectonic feature of the Upper Rhine Graben and the Biberach and Ravensburg areas, and, in particular, the Ziemetshausen area more than 200 km away, is most probably much too large to produce voluminous seismites in these areas. The East African Rift Valley is currently the largest seismically active rift system on Earth. Historical earthquakes and palaeoseismic events are estimated to have reached a maximum moment magnitude of M_W_ ~ 7.0^69^, an earthquake intensity sufficient to produce seismites within an area of ~ 100 km from the seismic source. Although the Biberach and Ravensburg sites could have been seismically affected by a very strong mid-Miocene earthquake with its hypocentre in SW Germany (e.g., the Hohenzollern- or /Lauchertgraben; Fig. 10), the Ziemetshausen area is still situated far beyond the sphere of influence (at least 50 km) from all seismic sources thought to have been active in the Middle Miocene. This makes the scenario of a tectonic or volcanic seismite-producing event in the study area very unlikely for the upper seismite horizon exposed in the Ziemetshausen sand pit. In contrast, the Ries and the Steinheim events both caused major seismic shaking and are both located only some 55 km away from this locality.

It is also noteworthy that the upper (Steinheim) seismite at Ziemetshausen, located only ~ 53 km SE of the Steinheim crater, is the thickest (4 to 5 m) and most distinctly developed upper seismite horizon in the entire study area, whereas at other localities (in the Hochgeländ area and at Dietenwengen sand pit) the upper seismite horizons is developed less distinctly and only has a thickness between 0.5 and 2 m. This indicates the seismic source for the upper seismite horizon was situated somewhere north of Ziemetshausen, and not in the Alps in the South or in any major tectonic structure west of this area. This agrees very well with the vertical extension and thickness of the clastic dikes that also decrease from North to South in the study area. Although direct evidence for the context between the Steinheim impact and the formation of the upper seismite horizon and clastic dikes (e.g. superimposed distal Steinheim ejecta on Steinheim seismite) still needs to be established, the number and quality of biostratigraphic, event stratigraphic, and geographic arguments provide a comfortable degree of certainty that the upper seismite horizon was most likely formed as a result of the Steinheim impact-earthquake.

### Growing evidence for two separate impact events

In summary, the recent findings related to the Ries and Steinheim impacts, including absolute impact ages^5,6^, high-resolution stratigraphic constraints aided by the presence of distal Ries ejecta in the field^3,14–16,27^, the differing biostratigraphic ages of the Ries and Steinheim crater lake deposits^1,3,33^, and the biostratigraphic correlation of the lower (Ries, transition MN 5/6) and upper (Steinheim, MN 6 to transition MN 6/7) earthquake-produced seismite horizons with the two source craters, respectively^3,16,27,31^, lead us to conclude that the Ries and Steinheim impact structures are the result of two temporally separate impact events in southern Germany, occurring only ~ 40 km and ~ 0.5 to 0.8 Myr apart. The Ries and Steinheim impact craters, therewith, extend the list of terrestrial false impact crater doublets including the West and East Clearwater Lake impact structures in Canada^70^ and the Suvasvesi North and South impact structures in Finland^71^, all of which had been considered of binary impact origin previously. Currently, only one crater pair on Earth remains (the Lockne and the Målingen impact structures, Middle Sweden) that is still regarded to be the result of a simultaneous double impact event^72^.

## Methods

### Field studies

Over the last three decades, horizons of distal Ries ejecta were systematically investigated in the NAFB^3,14–16^. We payed particular attention to ravines in the areas south of Ulm, Biberach and Ravensburg in SW Germany. Outcrops with soft-sediment deformation structures and clastic dikes and sills were partially exposed below and above the distal ejecta horizon along the flanks of the ravines after very heavy rainfall in winter 2018/2019 in the Biberach and Ravensburg area and were first discovered and investigated in spring 2019. The structures were excavated during various field campaigns from spring until winter 2019. We excavated and examined in detail the sandy foreland basin deposits over a vertical extension of up to 20 m along the flank of some ravines in the Hochgeländ area (e.g., ‘Tobel Oelhalde-Nord’ near Biberach) and over tens of metres laterally along the flanks of the ravines in the Ravensburg area and in an active sand pit near Dietenwengen. During various field campaigns in 2020, we discovered several new outcrops with Ries seismite overlain by distal Ries ejecta and dike swarms in the Hochgeländ plateau and near Ravensburg. In spring and summer 2021, as well as in spring 2022, we finally discovered outcrops with two separate seismite horizons in a ravine in the Hochgeländ plateau (Josefstobel), inspected and documented in detail. The former Ziemetshausen sand pit was inspected in detail in the years 2005 and 2008 by members of our group and/or by colleagues, mentioned as data source in the text and the acknowledgements.

### Estimated magnitude of impact earthquakes

Seismic efficiency (i.e., the fraction of the impactor's kinetic energy that is transformed into seismic wave energy) is thought to range between 10^−5^ and 10^−3^. Using a mean value of 10^−4^ for that efficiency^21,26^ (and references therein), an equation that correlates the impact energy with the resultant seismic magnitude (M_L_) was derived^21,26^:

1 $${\text{M}} = 0.67 \, \log_{10} {\text{E}}^{ - 5.87}$$

where M is the local (Richter) magnitude and E is the kinetic energy of the incoming projectile (E = half the projectile mass multiplied with the projectile's velocity squared, in Joules). Earthquake magnitudes calculated using that equation are only (geologically reasonable) approximations. Applying Eq. (1), the giant Chicxulub impact, for instance, (impact energy ~ 3.7 × 10^23^ J) that caused the mass extinction event at the K-Pg boundary generated a seismic pulse roughly equivalent to a moment magnitude M_W_ 10–11.5 earthquake^25^. The causal relation between the magnitude-distance relation of the formation of seismites in the form of clastic dikes and soft-sediment deformation caused by intense earthquake activity was reported for many regions on Earth^41,42,68^. Liquefaction and concomitant formation of seismites caused by meteoritic impact-induced earthquakes is preserved in the sedimentary record^16,17,25,28^ and can help to evaluate intensity of other impact-induced earthquakes. However, the impact earthquake magnitude-distance relationship for liquefaction effects in sediments has to be evaluated mainly from more recent large seismically-induced earthquakes and their distal dewatering effects reported in the literature^68^. For earthquake magnitudes given exclusively in local (Richter) scale magnitude M_L_ in the literature, we estimated M_W_ values (moment magnitude) based on existing M_L_ values. The moment magnitude (M_W_) and local (Richter scale) magnitude (M_L_) are roughly comparable between M_W_ ~ 3.5 and M_W_ ~ 7.0–7.5 for shallow earthquakes (depth < 33 km); at higher magnitudes saturation of M_L_ occurs and the pseudo-linear relationship is no longer valid. M_L_ values for the Ries and Steinheim impacts were calculated using well-established equations and impact energy values from the literature. In an additional step, we estimated moment magnitudes M_W_ from reported M_L_ values by comparing known M_L_ and M_W_ values for historical earthquakes (for detailed procedure and literature cited see^3^; Supplementary File). A range of typical M_L_ and M_W_ values for tectonic earthquakes and estimates for impact-triggered earthquakes is given in the supplementary Table 1 of an earlier study from our group^3^.

## Data Availability

All data generated or analysed during this study are included in this published article [and its supplementary information files].
